# Analysis of chromatin boundary activity in Drosophila cells

**DOI:** 10.1186/1471-2199-9-109

**Published:** 2008-12-11

**Authors:** Mo Li, Vladimir E Belozerov, Haini N Cai

**Affiliations:** 1 Department of Cellular Biology, University of Georgia, Athens GA, 30602, USA

## Abstract

**Background:**

Chromatin boundaries, also known as insulators, regulate gene activity by organizing active and repressive chromatin domains and modulate enhancer-promoter interactions. However, the mechanisms of boundary action are poorly understood, in part due to our limited knowledge about insulator proteins, and a shortage of standard assays by which diverse boundaries could be compared.

**Results:**

We report here the development of an enhancer-blocking assay for studying insulator activity in Drosophila cultured cells. We show that the activities of diverse Drosophila insulators including suHw, SF1, SF1b, Fab7 and Fab8 are supported in these cells. We further show that double stranded RNA (dsRNA)-mediated knockdown of SuHw and dCTCF factors disrupts the enhancer-blocking function of suHw and Fab8, respectively, thereby establishing the effectiveness of using RNA interference in our cell-based assay for probing insulator function.

**Conclusion:**

The novel boundary assay provides a quantitative and efficient method for analyzing insulator mechanism and can be further exploited in genome-wide RNAi screens for insulator components. It provides a useful tool that complements the transgenic and genetic approaches for studying this important class of regulatory elements.

## Background

Chromatin domain boundaries, also known as insulators, are important for the proper regulation of gene expression in a wide variety of organisms (for recent reviews of chromatin boundaries, see [1-8]). The best-known examples of chromatin boundary elements include scs and scs', which delimit the active chromatin domain of the Drosophila hsp70 genes during heatshock [9,10]. Other well-characterized boundaries include the yeast telomeric and silent mating type loci boundaries, which restrict the spread of repressive chromatin, and the mammalian ICR boundary, which modulates enhancer-promoter interactions in imprinted H19 and Igf2 loci [11-16]. Despite the diverse genomic contexts and different organismal origins, chromatin boundaries are characterized by either one or both of the following functional properties: their ability to block enhancer-promoter interactions when positioned interveningly (insulator activity, see [17-22]), and their ability to protect reporter genes from the transcriptional influences from the surrounding genome (barrier activity, [9,23-25]).

The mechanism of boundary activity remains poorly understood. This is partly due to our ignorance about their protein components, and a lack of systematic and comparative analyses of various insulator activities. Currently, boundary activities are often defined by assays that are unique to their organism of origin. For example, cell culture-based assays have been widely used to characterize vertebrate boundaries [21,24,26]. In contrast, characterization of many boundary elements in Drosophila were carried out in transgenic reporter assays [9,10,18-20,27-32]. Parallels were frequently drawn between activities defined in different assays and they could be misleading.

To begin addressing these problems, we developed a cell-based insulator assay to analyze and compare different boundary elements from Drosophila, the species where the most diverse collection of boundaries have been reported. The assay retains the key aspects of a P-element-based enhancer-blocking assay we previously used for investigating insulator function in transgenic Drosophila embryos [18,33]. It utilizes separate and clearly delineated enhancer and basal promoter modules, essential for testing enhancer-blocking activity. It contains divergently transcribed dual reporters, which provide a linked internal control against silencer effect and off-target effects. The use of GFP and RFP reporters facilitates the use of fluorescence-based quantification of enhancer-blocking activity. An important and unique feature is the use of P-element as the transgene backbone, which allows single or low copy number non-tandem genomic insertions of the assay transgenes in stable cell lines, providing a more suitable genomic and regulatory environment to study chromatin boundary function. We validated the novel assay with multiple Drosophila chromatin boundaries including the Gypsy insulator suHw element, the SF1, SF1b, Fab7 and Fab8 boundaries from the homeotic gene clusters. We further tested RNAi-mediated gene knock-down with the insulator assay and found that dsRNA against SuHw and dCTCF, two proteins essential for the function of suHw and Fab8, respectively, specifically disrupted the enhancer-blocking activity of these two insulators [34-36]. The system provides a rapid, efficient, and quantitative platform for comparing and analyzing diverse boundary elements, for dissecting boundary mechanism biochemically and for genome-wide RNAi screening of novel boundary components [37].

## Results

### An enhancer-blocking assay in cultured Drosophila cells

**Figure 1:**
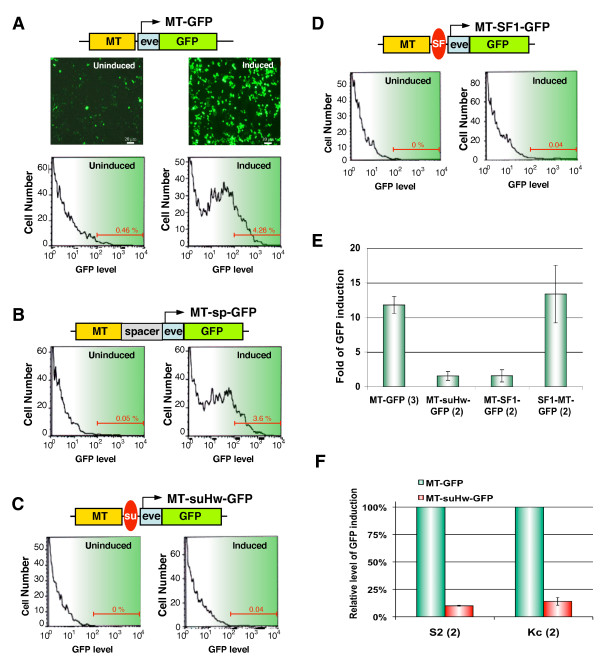
**Enhancer-mediated GFP activation is blocked by the suHw and the SF1 insulators in Drosophila S2 cells**. A. Induction of S2 cells containing the GFP transgene. Top: a diagram of MT-GFP: key regulatory components shown include: the MT enhancer (yellow), the evenskipped basal promoter (light blue) and the GFP reporter gene (green). Middle: fluorescence microscopy images of S2 cell containing MT-GFP before (left) and after (right) induction. Bottom: FACS histogram of uninduced (left) and induced (right) S2 cells. X-axes: log scale of GFP level; Y-axes: cells number at indicated GFP level. Red bar: percentage of total cells with GFP level above 10^2^. B. Induction of S2 containing MT-sp-GFP (grey box). Bottom: FACS histogram of uninduced (left) and induced (right) S2 cells. C-D. GFP induction in S2 cells containing enhancer-blocking transgenes. Top: a diagram of MT-suHw-GFP (C) or MT- SF1-GFP (D) transgenes. Insulator elements are represented by the red ovals. Bottom: FACS histogram of uninduced (left) and induced (right) S2 cells. E. Quantitation of GFP induction (I) in S2 cells transfected with insulator-containing transgenes. Number of replicates for each experiment is shown in parentheses. See methods for calculation of I and standard error of mean (SEM). F. Comparison of enhancer-blocking activity of suHw in S2 and Kc cells. Bar graph shows percentage of GFP induction in S2 (left) or Kc (right) cells transfected with MT-suHw-GFP (red bars) and MT-GFP (green bars). Transfection and induction were done in parallel.

An important consideration in designing a transgene for testing enhancer-blocking activity is the selection of a pair of clearly delineated and well-matched enhancer and promoter. For the promoter, we tested the basal promoters of the hsp70 and evenskipped (eve) genes. The eve basal promoter contains a 42-bp upstream sequence and a canonical TATA box [38]. It exhibits low basal activity on several reporter genes but responds robustly to a variety of enhancers in transgenic Drosophila [39-41]. The hsp70 basal promoter has been used widely to drive various reporter genes, such as GFP and RFP [42]. For the enhancer we selected a Cu^2+^-inducible metal response enhancer from the metallothionein gene (MT, [43]). The enhancer and promoter pair was combined with a GFP reporter in a P-element backbone (see map of MT-eve-GFP, Figure 1A) and introduced into Drosophila S2 cells via transient transfection. Addition of 1 mM Cu^2+ ^in the media resulted in strong induction of the GFP reporter (upper panels, Figure 1A). Fluorescence-Activated Cell Sorting (FACS) showed that this corresponded with a 10-fold increase in the frequency of GFP positive cells when compared with the no-induction control (bottom panels, Figure 1A, Figure 1E). GFP induction was not observed in a control transfection in which the MT enhancer was absent (data not shown). These results indicate that the GFP expression is a good indicator for the activity of the MT enhancer. GFP was also strongly induced from a transgene containing a spacer DNA between MT and eve-GFP (MT-sp-GFP, Figure 1B). Since the effect of an upstream enhancer could be mediated from either the forward (MT -> spacer -> eve-GFP) orientation, or the reverse (MT -> P-vector -> GFP-eve promoter) orientation along the circular plasmid, we linearized the transgene at a position distal to the enhancer and compared the GFP induction using the linearized plasmids. We found little difference in the efficiency of GFP induction between the two DNAs, suggesting that either the distance between MT and the eve promoter in the reverse orientation (~5 kb) is prohibitory, or there might be insulator-like activities in the vector backbone (data not shown). Next, we tested the ability of the suHw element, a well-characterized Drosophila boundary element from the Gypsy retrotransposon, to block the MT enhancer. The 340 bp suHw element inserted between MT and the eve-GFP fusion reporter almost completely blocked the Cu-mediated induction of the GFP reporter (MT-suHw-GFP, Figure 1C). A similar enhancer-blocking effect was also seen when the SF1 chromatin boundary, a 2.4-kb DNA element from the Drosophila Antennapedia complex, was placed between the MT enhancer and the GFP reporter (Figure 1E, [31]). In contrast, the same SF1 insulator, when placed upstream of the MT enhancer, had little effect on GFP induction (Figure 1E). These results show that the inhibitory effect of the insulators depends on their intervening position between the enhancer and the promoter, a key characteristic that distinguishes insulators from silencers. We further tested the insulator activity in another commonly used Drosophila cell line, the Kc cells [44,45]. We found the enhancer-blocking activity of the suHw element in Kc cells to be comparable to that observed in the S2 cells (Figure 1F). These results indicate that the suHw boundary is active in these cultured Drosophila cells.

Stably transfected cells contain integrated transgenes in a chromosomal environment, which more closely resembles the native chromatin environment of boundary function. Further, P-element-based transposition has been shown to produce predominantly low copy number solitary insertions in stably transfected Drosophila cells [46]. Such an arrangement provides a more native regulatory environment to study insulator function without triggering homology-induced transgene silencing observed in tandem transgene arrangement, or altering the regulatory stoichiometry of the endogenous chromatin boundary system, which may contain up to hundreds of boundary sites dispersed in the genome [47-54]. In addition, stable cell lines allow careful calibration of the transgene behavior for detecting subtle changes in boundary activity, and provide consistent cell sources for biochemical studies and large-scale cell-based screens. We tested the enhancer-blocking assay in stably transfected S2 cells. In transfections using the reporter transgenes alone, frequency of GFP positive cells reduced from 12–25% at two days post transfection to 0.02–0.1% 4–5 weeks after transfection. We co-transfected S2 cells with GFP transgene and pTurbo, a P element transposase encoding plasmid, at 10:1 and 100:1 weight ratios. We found that P-transposase dramatically improved the frequency of stable integration of GFP reporter transgene in unselected cell populations. For example, the mean frequency of GFP positive cells is 6.5% 25–42 days post transfection (replicate n = 3), which is much higher than those without pTurbo. Although the mean induced GFP level is lower than transiently transfected cells due to the reduction in the transgene copy number, we still observed a 3–4 fold higher GFP induction in cells containing MT-GFP than those containing MT-suHw-GFP in unselected cell populations. These results indicate that the suHw insulator is active as integrated in these cultured Drosophila cells.

### Dual-reporter assay for boundary activity

**Figure 2:**
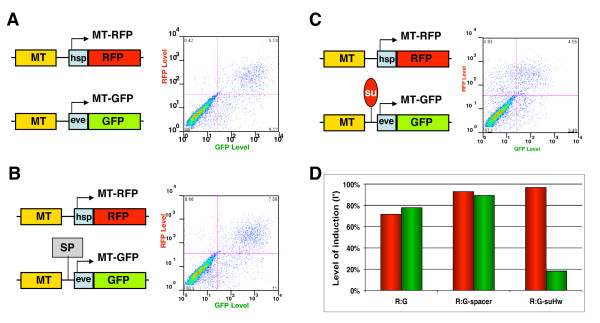
**Enhancer-blocking assay using two reporter transgenes**. Reporter expression in S2 cells cotransfected with separate RFP and GFP transgenes. A. Left, diagrams of MT-RFP (top) and MT-GFP (bottom). Right, FACS chart of GFP and RFP induction in S2 cells cotransfected with both transgenes. Level of GFP or RFP is shown in logarithm scale (Y or X axis, respectively). The lower-left quadrant contains cell with both GFP and RFP levels <80 (double-negative cells); and the top-right quadrant contains cells with both GFP and RFP levels >80. Percentage of cells indicated at the corner in each quadrant. B. Induction of S2 cells cotransfected with MT-RFP and MT-sp-GFP, which contains a spacer (grey box) between MT and the eve promoter. Right: FACS chart of GFP and RFP induction in S2 cells cotransfected with both plasmids. C. Induction of S2 cells cotransfected with MT-RFP and the MT-suHw-GFP, which contains the 340-bp suHw element (red oval) between MT and the eve promoter. Right: FACS chart of GFP and RFP induction in S2 cells cotransfected with both plasmids. D. Relative induction level (I') of the RFP (red bars) and GFP (green bars) in cotransfection experiments in Panels A-C, see Methods).

In order to assay for insulator activity, it is important to control for changes in the non-insulator components of the assay system that could affect reporter expression. An internal reference reporter within the same cell allows rapid and quantitative assessment of enhancer-blocking activity, especially in high throughput applications. We generated a control transgene containing the dsRed fluorescent protein (RFP) gene driven from the hsp70 promoter and the MT enhancer (MT-RFP, Figure 2A[42,55]). Cotransfection of the MT-GFP and MT-RFP plasmids produced cells doubly positive for GFP and RFP upon induction (the upper right quadrant, Figure 2A, [R:G] in Figure 2D). The ratio between the induced levels of GFP and RFP did not change when MT-RFP was cotransfected with MT-sp-GFP, which contains a spacer DNA between the MT enhancer and the eve promoter (Figure 2B, [R:G-spacer] in Figure 2D). However, when MT-RFP was cotransfected with MT-suHw-GFP, a transgene containing the suHw element between the MT and the eve-GFP reporter, the level of GFP was dramatically reduced compared to that of RFP (Figure 2C, [R:G-suHw] in Figure 2D). We concluded that the RFP transgene serves as an independent readout for the activity of the MT enhancer and the state of transcriptional activity in the cell. The ratio between the induced level of GFP and RFP is a good indicator of the boundary activity.

**Figure 3:**
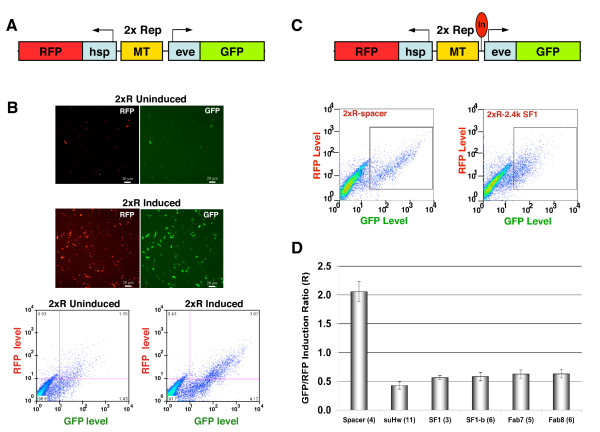
**Enhancer-blocking assay using a dual-reporter transgene**. Reporter expression in S2 cells transfected with a single 2xR dual reporter transgene. A. A diagram of the 2xR dualreporter transgene vector. B. Top: RFP and GFP expression ofS2 cell containing 2xR before (top) and after (middle) induction. Bottom: FACS chart of 2xR-transfected S2 cells before (left) andafter (right panel) induction. C. Top: a diagram of 2xR transgene with a test insulator (In, red oval) inserted between MTand the eve-GFP reporter. Bottom: FACS chart of induced S2 cells containing 2xR-spacer (left) and 2xR-SF1 (right). Insets containing double positive cells are shown at identical position. D. Ratio (R) between GFP and RFP induction in S2 cells containing 2xR-suHw, SF1, SF1b, Fab7, and Fab8, respectively. Number of replicates for each experiment is shown in parentheses. Standard error of mean (SEM) is indicated.

Next, we created a dual-reporter construct that contains both GFP and RFP reporters in divergently transcribed orientation driven by the eve promoter and the hsp70 basal promoter, respectively. The MT enhancer is inserted between the two promoters (2xR, Figure 3A). This transgene allows both GFP and RFP reporters to be present in the same cells, with the same copy number and at the same genomic location, thereby providing a necessary control for interpreting insulator activity in stably transfected cells. Cells transiently transfected with the 2xR dual reporter transgene showed strong induction of both GFP and RFP expression (Figure 3B). The magnitude of GFP induction appeared slightly higher than that of RFP under the same detection and compensation conditions used in the cotransfection (Figure 3B). We also noticed that the level of GFP induction was slightly reduced by the insertion of a spacer DNA (Figure 3C, and 3D, see methods for calculation of ratio of GFP and RFP induction (R)). However, a known insulator SF1 inserted between the MT enhancer and the eve-GFP reporter caused a significantly greater reduction in the GFP/RFP induction ratio than the spacer control (Figure 3C, D).

### Diverse Drosophila boundaries function in S2 cell assay

In order to establish the general efficacy of the cell-based insulator assay, we tested several Drosophila boundary elements in the dual reporter assay. They include suHw, SF1, SF1b, a sub-element of SF1, Fab7 and Fab8 (Figure 3D, [27,31,36,56-58]). In transiently transfected S2 cells all tested insulators effectively reduced the MT activation of the GFP reporter when compared with that of RFP (Figure 3D). We also tested Fab8 inserted in both orientations between MT and GFP, and both significantly blocked the MT enhancer (forward orientation: R = 31% of no insulator control, reverse orientation: R = 42%). These results indicate that the 2xR transgene is suitable assay vector for diverse insulators.

### Probing boundary function using RNAi

Although Drosophila has the most diverse insulator proteins identified so far, functional tests of all known insulator proteins on all insulator elements have not been systematically carried out. A goal of developing a cell-based insulator assay is to use RNAi-mediated gene knockdown to identify and characterize protein components of insulators. Double-stranded RNA (dsRNA) induces powerful interference of gene activity in Drosophila in a cell-autonomous and isoform-specific fashion. It has been the predominant agent for RNA-interference both in vivo and in cell culture studies in Drosophila [59]. Our insulator assay supports the activity of diverse Drosophila insulators in a more native regulatory environment and should provide a more suitable system for studying the transacting components of boundary function.

**Figure 4:**
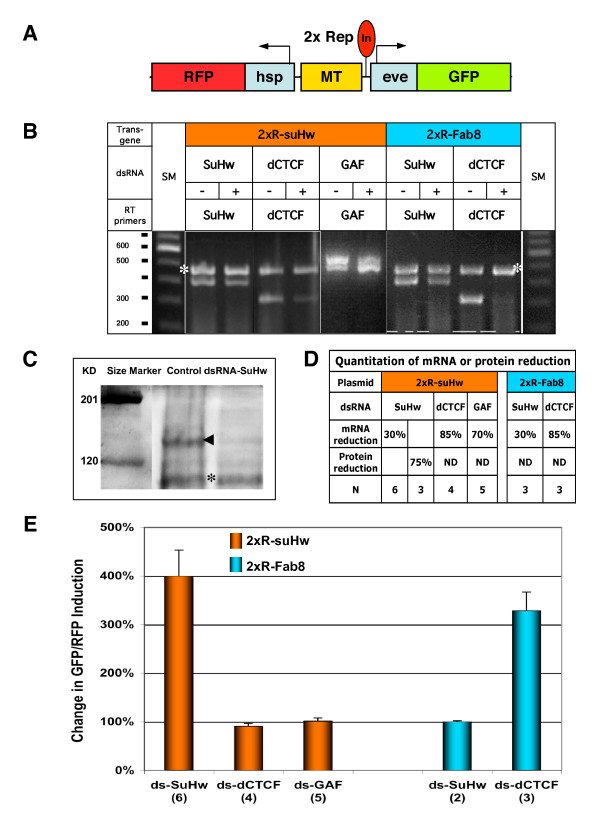
**RNAi -mediated disruption of insulator function in the S2 assay**. A. Diagram of 2xR transgenes used in RNAi knockdown tests. B. RT-PCR assessment of SuHw, GAF and dCTCF transcript level in S2 cells containing 2xR-insulator transgenes. Transgenes used in transfection are indicated on top of the panel. Double-strand RNA (dsRNA) used in knockdown treatment and mRNA-specific primers used in RT-PCR reactions are indicated on the left. S2 cells not treated (-, left lanes) or treated (+, right lanes) with dsRNA were used in RT-PCR using gene-specific primers and Actin 88f primers. The asterisk indicates the expected actin product at 370 bp. C. Western blot analysis of SuHw protein level in S2 cells. Left lane, molecular weight standard in kilodalton (KD); middle lane, untreated cells; and right, dsRNA-SuHw-treated cells. Arrowhead points to the position of SuHw at ~145 KD. Asterisk indicates a non-specific band reactive to the antibody. D. Summary of mRNA and protein reduction in the RNAi-mediated knockdown. N indicates the number of replicate of RT-PCR used in the assessment. E. Changes in GFP/RFP ratio as a result of knockdown (untreated cell = 100%). The dsRNA used in the knockdown is indicated below the bar graph. Number of replicates is indicated in parentheses.

We first tested the effect of RNAi-mediated gene knockdown on the suHw insulator. SuHw, a zinc-finger protein, is critical for the suHw insulator activity [34,35]. S2 cells transiently transfected with 2xR-suHw transgene were incubated for 96 hours with culture media containing dsRNAs against SuHw (Figure 4, [34,35,60]). These cells were then induced by Cu^2+ ^and analyzed for reporter expression. To assess the extent of SuHw knockdown, duplex semi-quantitative RT-PCR were performed using primers against the mRNAs of SuHw, and those of Actin 88F as an internal control (left panel, Figure 4B, D). Cells treated with dsRNA-SuHw showed a moderate reduction in the level of SuHw mRNA when compared to untreated control cells (Figure 4B, also see Figure 4D for quantitation of SuHw mRNA knockdown). To validate the knockdown at protein level, we used Western blot to compare the SuHw protein level in dsRNA-treated cells to that in untreated control cells. We observed a significant reduction of SuHw in the dsRNA-treated cells (Figure 4C, D for quantitation of SuHw protein knockdown). Such differential knockdown in mRNA and protein levels has been previously observed in RNAi experiments [61]. The treatment of dsRNA-SuHw coincided with a dramatic increase in the GFP/RFP ratio when compared with the untreated control (Figure 4E). This change is consistent with the key role SuHw plays in the enhancer-blocking activity of the insulator. Loss of insulator activity was not observed when these cells were treated with dsRNAs against dCTCF or GAF, two other proteins implicated in the function of Drosophila Fab8 and SF1/Fab7 insulators, respectively (Figure 4B, D, [31,36,58]). We also tested SuHw knockdown in cells transfected with Fab8-containing transgenes. Comparable reduction in the SuHw mRNA resulted in little or no change in the GFP/RFP ratio in these cells (Figure 4B, D–E). These results demonstrate the specificity of the SuHw protein to the suHw insulator. It is important to note that the dsRNA-treated cells, including those used in SuHw, dCTCF or GAF knockdowns, appeared normal and indistinguishable, other than the level of reporter expression, from the untreated control cells during the duration of the experiment.

We next focused on the role of dCTCF, a recently identified component of the Drosophila Fab8 insulator [36]. The dCTCF gene encodes the ortholog of the vertebrate CTCF, which is required for the enhancer-blocking activity of almost all vertebrate boundaries known to date [2,36,62-64]. It contains multiple zinc fingers, a shared structural feature with the SuHw protein, and was found to localize at a large numbers of sites in the Drosophila genome [50,52,65]. S2 cells were transfected with dual-reporter transgene containing the Fab8 insulator and treated with dsRNA-dCTCF. Reporter induction was examined in treated cells and compared with untreated controls. Treatment of dsRNA-dCTCF elicited a severe reduction of the dCTCF mRNA (Figure 4B, D). This resulted in a dramatic loss of insulator activity of Fab8, shown by the 230% increase in the GFP/RFP induction ratio compared to the untreated control (Figure 4E). However, a similar reduction in dCTCF resulted in little or no effect on the enhancer-blocking function of suHw (Figure 4B, D–E). Our results indicate that dCTCF is likely to be a dedicated component of the Fab8 class insulators. The efficient knockdown of the dCTCF-dependent insulators in the Drosophila cell could be utilized as a model for studying the vertebrate CTCF-dependent boundary pathways. These results further suggest that multiple independent classes of boundaries, exemplified by the Gypsy (suHw) type with the dedicated SuHw factor, the SF1 or Fab7 type, which depends on the Drosophila GAF protein, and the Fab8 type, facilitated by dCTCF, carry out boundary functions in Drosophila [31,35,36,58,66,67].

## Discussion

We have developed a novel cell-based assay for studying insulator function in Drosophila. We show that despite their diverse genomic origins and distinct cis- and trans- components, the Drosophila suHw, SF1, Fab7 and Fab8 elements function as potent enhancer-blockers in the Drosophila cells. This finding suggests that chromatin boundary represents a basic cell function that is shared by diverse tissues. We further combined the cell-based insulator assay with RNAi-mediated gene knockdown to systematically test the requirement of SuHw and dCTCF in the function of several Drosophila insulators. We showed that RNAi-mediated knockdown of SuHw and dCTCF specifically disrupted the function of the suHw and Fab8 boundaries, respectively, thereby validating the functional specificity of the assay. Our results suggest that multiple independent pathways in Drosophila mediate insulator function [31,35,36,58,66,67]. This is in contrast with the pivotal role the CTCF protein plays in the enhancer-blocking activities in vertebrates.

Cell culture assays have several important advantages that complement studies using in vivo system. The homogeneous cell populations in these assays can be used in biochemical and cell biological analyses. They allow more efficient and quantitative assessment of reporter readout from a large number of individual cells. Insulator activity has previously been demonstrated in Drosophila cells, our system has improved the assay with several novel features [68,69]. First is the use of P-element-based transgene vector, which is known to mediate single to low copy number, non-tandem genomic integration of the assay transgenes [46]. This would provide more native genomic and regulatory environment for studying chromatin boundary function. Large numbers of stably transfected cells with randomly integrated transgenes also provide a broader sampling of the genomic environment, a feature that can be exploited to examine boundary activity in blocking chromosomal position effect. The second improvement is the use of divergently transcribed dual reporters, which provides a linked readout to control for the "off-targets" effects on the non-insulator components in the assay system, such as enhancers, promoters, reporters, the state of general transcription or other cellular functions that impact the reporter readout. It should also provide an important control for the chromosomal position effect near the transgene integration site in stably transfected cells. The use of fluorescent protein reporters further allows rapid and quantitative FACS assessment of the enhancer-blocking activity, a feature particular important in high-throughput applications. We have now established the activity of multiple Drosophila insulators and the efficiency of RNAi-mediated gene knockdown in our assay, which should facilitate biochemical dissection of insulator function and genome-wide high throughput RNAi screens for novel boundary components [37].

As most cell-based systems, the enhancer-blocking assay is limited in its application by potential tissue or developmental stage incompatibilities of the insulator and the cell. Studies have suggested that certain chromatin boundaries, such as Fab7 and SF1, are composed of distinct insulator activities that function in different tissues and/or developmental stages [70], Roy and Cai, unpublished). Although we have documented the functionality of several Drosophila insulators in S2 and Kc cells, both derived from embryonic cell lineages, other insulators may not function in these two cell lines [71]. In addition, cultured cells may have, over the course of many passages, lost the physiological stoichiometry of relevant DNA or protein components, resulting in impaired function of certain insulators. Furthermore, the dynamic regulation of insulator activity in response to developmental and physiological cues would depend on the context of the whole animal. Therefore, the cell-based insulator assay we presented here provides a useful tool that complements the transgenic and genetic approaches for studying this important class of regulatory elements.

## Methods

### Construction of DNA plasmids used in S2 and transgenic embryo insulator assays

The EGFP open reading frames were amplified by PCR using pEGFP-N3 (Clonetech) as templates, and subsequently inserted between BamHI and PstI sites replacing the lacZ ORF in the pCAsPeR-eb-lacZ construct [38,40]. To make the double reporter construct (pCA-2xR), the DNA fragment containing the 128 bp hsp70 promoter fused to the RFP coding region was amplified from pRed Stinger (kindly provided by S. Barolo), subcloned into pCRII TOPO vector and then inserted between the NsiI and EcoRV sites of pCA-EB replacing the mini-white gene. The Metallothionein (MT) enhancer was cloned by PCR. Primer sequences will be provided upon request (below same). The MT enhancer was placed into pCA-eve-GFP upstream from the promoter or between the divergently transcribed reporters in pCA-2xR. The spacer element, SF-1, SF-1/b, suHw, Fab7 and Fab8 were purified as NotI fragments and inserted into a NotI site between the MT enhancer and eve-GFP in the single- and double-reporter constructs.

### S2 cell culture and transfection

Drosophila Schneider's Line 2 (S2) cells were maintained in HyQ SFX-Insect serum-free medium (HyClone) at 25°C. Cells were sub-cultured every 10 days. The DNAs used for transfection were prepared using the Qiagen Plasmid Mini Kit. For transfection S2 cells were sub-cultured 3–5 days before transfection, 5 × 10^5 ^cells in 1 ml medium were aliquoted into each well of a 12-well plate. After cells had attached to the bottom of the well, they were gently washed once with 1 ml of fresh medium and soaked in 0.5 ml of transfection cocktail (1 μg of assay construct and 2.5 μl of Cellfectin reagent in 0.5 ml medium). For stable transfection, pTurbo plasmid containing the P-element transposase was mixed with the assay construction at a ratio of 1 to 10. The transfection cocktail was replaced with fresh medium after 5 hour of incubation. Cells were normally induced with 1 mM CuSO_4 _24 hours after transfection except in the RNAi experiments.

### Imaging and flow cytometry

Fluorescence microscopy and flow cytometry analysis were done 24 hours after induction. Images of the cells were taken with an Olympus DP10 digital camera attached to a Zeiss Axioplan 2 fluorescence microscope. Fluorescence Activated Cell Sorting was performed using FACSCalibur flow cytometer (Becton Dickinson Immunocytometry Systems). Briefly, cells were wash off the plate, spun down and resuspended in sterile PBS. Fifty thousand cells were analyzed for each sample. Fluorescence was excited at 488 nm. The photomultiplier detection voltages were set at 400 V for FL1 and 375 V for FL2. Data analysis was done using the Flojo software. Green fluorescence was detected with FL1 530/30 BP filter; red fluorescence was detected with FL2 585/42 BP filter. For MT-GFP transgenes, the fold of induction in Figure 1E is calculated as below: I = [% of GFP positive cells (>10) × mean GFP fluorescence]_induced_/[% of GFP positive cells (>10) × mean GFP fluorescence]_uninduced_. The relative level of induction of the reporter genes in cotransfection experiments in Figure 2D is calculated as below: I' = (i/i_max_) × 100%, (i = positive cells % × positive cells mean fluorescence]_induced_/[positive cells % × positive cells mean fluorescence]_uninduced_, and i_max_. is the nearest high integer if the highest i value of all cotransfection experiments. The ratio of relative level of induction (R) of the two reporter genes in the dual reporter experiments in Figure 3D and Figure 4E is calculated as below: R = {[GFP cells % × GFP cells mean fluorescence]_induced_/[GFP cells % × GFP cells mean fluorescence]_uninduced_}/{[RFP cells % × RFP cells mean fluorescence]_induced_/[RFP cells % × RFP cells mean fluorescence]_uninduced_}. For all quantitation graphs, the error bar is calculated as: SEM = standard deviation (SD)/square root [replicate number (N)].

### RNAi and RT-PCR

To synthesize double-strand RNA for the RNA interference experiment, linearized cDNA plasmid or PCR product for the target proteins were used as templates for in vitro transcription reaction. The SP6 and T7 MEGAscript transcription kits (Ambion) were used to generate sense and anti-sense RNA strands. The two strands were then annealed and quantitated before use. The dsRNA-SuHw was synthesized from a cDNA clone generously provided by Jim Kodonaga, or a PCR fragment amplified with the primers 5'AGGAAAAGAAGGGCAAGCTGC3' and 5'AGCATATGTCCTTCTTCTCC3'. The dsRNA-GAF was synthesized from a cDNA clone generously provided by Sally Elgin, or a PCR fragment amplified with the primers 5'TAATACGACTCACTATAGGGACCAAGACCAACTGATTGCC3' and 5'TAATACGACTCACTATAGGGCCTTTTGTCCTTCGCTCTTG3'. The dCTCF cDNA plasmid was purchased from ATCC. For RNAi experiment, cell transfection was performed as described above. 24 hours after transfection, cells were washed off the plates and aliquoted into 12-well plates at 3 × 10^5 ^cells/well. Double-strand RNA was delivered to the cells either by transfection using Cellfectin reagent or by soaking the cells in ds-RNA containing medium. Cells were induced by addition of 1 mM Cu^2+ ^72–96 hours after RNAi treatment. 24 hours after induction, cells were harvested for FACS analysis and total RNA extraction using TRIZOL reagent (invitrogen). RT-PCR analysis was performed to assess the effectiveness of the RNAi knockdown. The isolated RNAs were used as templates in RT-PCR reactions using the Qiagen OneStep RT-PCR kit. The following primers were used to detect transcripts: 5'GGAAAACACAGCCCGAAACA3' and 5'CCTCATCCGTCAGCTGCTCT3' for Su(Hw); 5'TGTCACAATGGTCTGCTGTTGT3', 5'GTATCGGCAATCCAATTGTTG3' for GAF;; 5'AGTACAGCCACCAATAAATCCATC3', 5'CTTCGTCTACGGTATAGTCCGACA3' for dCTCF; 5'GATGGTGTCTCCCACACCGT3' and 5'CGATCGGCAATACCAGGGT3' for actin 88F. A semi-quantitative multiplex RT-PCR was performed for each treatment with the primers for the transcript of the target gene and the primers for actin 88F as an internal control. The ratio of target gene primers to the actin 88F primers was determined empirically. Equal weight of RNA template was used for all RT-PCR reactions. Changes in GFP/RFP ratio due to the RNAi knockdown is calculated as below: C = {[GFP cells % × GFP cells mean fluorescence]_RNAi treated_/[GFP cells % × GFP cells mean fluorescence]_untreated_}/{[RFP cells % × RFP cells mean fluorescence]_RNAi treated_/[RFP cells % × RFP cells mean fluorescence]_untreated_}.

### Western blot analysis of the SuHw protein

S2 cell transiently transfected with 2xR-suHw transgene and treated for 72–96 hours either with dsRNA-SuHw or control media. Half million cells were harvested, lysed by boiling in 4× sample buffer and fractionated on 8% Polyacrylamide-Bis gel with prestained molecular weight standard. Proteins were transferred and immobilized to PVDF membrane and incubated with a primary rabbit anti-SuHw antibody generated against a 20-aminio acid-polypeptides (KFSALVALKKHRRYHTGEKP). The antibody was preabsorbed against 0–18 h Drosophila embryos before use. A secondary anti-rabbit-AP conjugate is from Sigma. Colorimetric visualization of SuHw protein were done using BCIP/NBT tablet (Sigma). Digital image analyses were performed using the ImageJ software.

## Authors' contributions

VB designed and carried out the single-reporter insulator assays in S2 cells and cotransfection experiments in S2 cells. ML designed and carried out the insulator assays in Kc cells, 2X-reporter insulator assays and RNA interference studies in S2 cells. HC participated in the design of the study, data analyses and discussion and manuscript preparation. All authors read and approved the final manuscript.
